# Perinatal outcomes in a South Asian setting with high rates of low birth weight

**DOI:** 10.1186/1471-2393-9-5

**Published:** 2009-02-09

**Authors:** Kuryan George, Jasmin Prasad, Daisy Singh, Shanthidani Minz, David S Albert, Jayaprakash Muliyil, K S Joseph, Jyothi Jayaraman, Michael S Kramer

**Affiliations:** 1 Department of Community Health, Christian Medical College, Vellore, India; 2 Departments of Obstetrics and Gynaecology and Pediatrics, Dalhousie University, the IWK Health Centre, Halifax, Nova Scotia, Canada; 3 St. Margaret's Bay Medical Centre, Halifax, Nova Scotia, Canada; 4 Departments of Pediatrics, Epidemiology and Biostatistics, McGill University, Montreal, Quebec, Canada

## Abstract

**Background:**

It is unclear whether the high rates of low birth weight in South Asia are due to poor fetal growth or short pregnancy duration. Also, it is not known whether the traditional focus on preventing low birth weight has been successful. We addressed these and related issues by studying births in Kaniyambadi, South India, with births from Nova Scotia, Canada serving as a reference.

**Methods:**

Population-based data for 1986 to 2005 were obtained from the birth database of the Community Health and Development program in Kaniyambadi and from the Nova Scotia Atlee Perinatal Database. Menstrual dates were used to obtain comparable information on gestational age. Small-for-gestational age (SGA) live births were identified using both a recent Canadian and an older Indian fetal growth standard.

**Results:**

The low birth weight and preterm birth rates were 17.0% versus 5.5% and 12.3% versus 6.9% in Kaniyambadi and Nova Scotia, respectively. SGA rates were 46.9% in Kaniyambadi and 7.5% in Nova Scotia when the Canadian fetal growth standard was used to define SGA and 6.7% in Kaniyambadi and < 1% in Nova Scotia when the Indian standard was used. In Kaniyambadi, low birth weight, preterm birth and perinatal mortality rates did not decrease between 1990 and 2005. SGA rates in Kaniyambadi declined significantly when SGA was based on the Indian standard but not when it was based on the Canadian standard. Maternal mortality rates fell by 85% (95% confidence interval 57% to 95%) in Kaniyambadi between 1986–90 and 2001–05. Perinatal mortality rates were 11.7 and 2.6 per 1,000 total births and cesarean delivery rates were 6.0% and 20.9% among live births ≥ 2,500 g in Kaniyambadi and Nova Scotia, respectively.

**Conclusion:**

High rates of fetal growth restriction and relatively high rates of preterm birth are responsible for the high rates of low birth weight in South Asia. Increased emphasis is required on health services that address the morbidity and mortality in all birth weight categories.

## Background

Several unique problems beset perinatal health in less industrialized countries including high rates of low birth weight (< 2,500 g) and infant death. According to UNICEF [1], low birth weight rates were 7%, 16% and 19%, respectively, in industrialized, developing and least developed countries in 2005. Of the approximately 20 million low birth weight infants born in 2005, more than half were born in South Asia, which has a low birth weight rate of 29% [1]. Estimated infant death rates in industrialized, developing and least developed countries in 2005 were 6, 83, and 153 per 1,000 live births, respectively [1].

The substantial burden of illness notwithstanding, perinatal health in less industrialized countries has received inadequate attention in the medical literature [2,3]. For instance, the relative frequency of low birth weight components, namely, preterm birth and fetal growth restriction, remains to be clarified. Some studies [4,5] show that the high rates of low birth weight in developing countries occur because of poor fetal growth, whereas other studies suggest that preterm birth (< 37 completed weeks) rates are high as well [6,7]. This is not merely an academic issue; from both an etiologic and prognostic viewpoint, low birth weight due to fetal growth restriction is very different from low birth weight due to a short pregnancy duration [8]. Thus, the distinction has important implications for developing and testing preventive interventions.

Another critical issue relates to public health policy for addressing perinatal morbidity and mortality in such settings. Whereas public health efforts have traditionally focused on preventing low birth weight [9-11], empirical evidence from both industrialized and less industrialized countries shows that substantial reductions in infant mortality rates in recent decades have occurred despite the absence of any reduction, and even simultaneous increases in low birth weight rates [12-14]. This suggests that targeting support for infrastructure and trained manpower to deal with the consequences of low birth weight may be a more effective policy option.

We attempted to answer these questions using population-based data from Kaniyambadi, a rural area in South India, where a community-based maternal and child health program has operated for over 2 decades. We assessed temporal trends in low birth weight, preterm birth, small-for-gestational age (SGA) birth, and perinatal and maternal mortality from 1986 to 2005. To provide an industrialized country context for examining absolute rates and temporal trends, we also analyzed similar population-based data from Nova Scotia, Canada.

## Methods

Kaniyambadi comprises a mostly rural population of about 112,000 (2008) in 80 villages of Tamil Nadu, South India. In addition to the regular health care offered by the government, primary and secondary health care services for these villages have been provided by the Community Health and Development (CHAD) program of Christian Medical College, Vellore, for over 50 years [15,16]. Basic health care in the villages is provided by part time community health workers under the supervision of health aides, who in turn are supervised by community health nurses. Monthly mobile clinics are conducted in each village by a doctor-led team and offer antenatal, immunization and other clinic-based services. High-risk pregnancies identified at the mobile clinics are referred to the CHAD hospital high-risk weekly clinic. Residents of Kaniyambadi access free hospital services from government institutions (primary health centre and a recently opened medical school hospital, both in Kaniyambadi, and the district hospital located in the nearby town) and also paid services from private hospitals, including the CHAD Hospital (located within Kaniyambadi and offering subsidized services to those with a lower socioeconomic status), and other private facilities in the nearby town (including Christian Medical College and Hospital, a tertiary care facility).

The CHAD program in Kaniyambadi includes a health information system, initiated in 1986 and described in detail elsewhere [17]. Surveillance of perinatal processes and outcomes is monitored thought the same four-tiered system that delivers health services. Kaniyambadi is divided into regions with specific personnel in charge of health services and surveillance. Every week the community health workers report (orally) to the health aide about pregnancies, deliveries, births, deaths, morbidity, marriages, immunisation and couples eligible for contraception in the village. This information is tabulated by the health aide on standardized forms and verified by the nurse on her fortnightly visit to the village and subsequently by the area doctor. Information on migration into and out of the villages is also obtained. The reports provided by the health aide are entered into a computerized database, which provides bi-weekly outputs to health aides (regarding pregnant women due for tetanus immunization, children due for immunization, etc) and monthly and annual reports to managers. Completeness of birth information is assured because the community health workers are resident in the villages and because of the frequent visits of CHAD program personnel to the villages in Kaniyambadi. Since its inception, this system and infrastructure has provided information for several studies (selected list [18-24]). The study included information on all births in Kaniyambadi from 1986 to 2005.

Information on all births in Nova Scotia was obtained from the Nova Scotia Atlee Perinatal Database. This database contains detailed information on births to Nova Scotia residents and is abstracted from antenatal and medical charts by trained personnel using standardized forms. For the years 1986 and 1987, the database included information on all births in Halifax county and most but not all births in the rest of Nova Scotia. All births in Nova Scotia were included in the database from 1988 onwards.

Since the only estimates of gestational age in the Kaniyambadi database were those based on the date of the last menstrual period, menstrual dates were used to estimate gestational age in both Kaniyambadi and Nova Scotia. Analyses were restricted to live births and stillbirths with a calculated gestational age between 20 and 50 completed weeks or a birth weight ≥ 400 g. Rates of low birth weight (per 100 live births) and trends by 2-year period were examined; the rate in 2004–05 was contrasted with the rate in 1986–87 using percent declines and 95% confidence intervals, while the statistical significance of temporal trends was assessed using a Chi-square for linear trend in proportions. Rates of preterm birth (per 100 live births), small-for-gestational age (per 100 live births), stillbirth (per 1,000 total births), early neonatal death (per 1,000 live births), perinatal mortality (per 1,000 total births) and maternal mortality (per 100,000 live births) and temporal changes were assessed using the same methods. Small-for-gestational age live births were identified using the 10^th ^percentile cut-off of a Canadian fetal growth standard [25]. We also estimated rates of small-for-gestational age using an Indian fetal growth standard, with the 10^th ^percentile value assumed to be 1.28 standard deviations less than the mean birth weight for gestational age [26]. Sensitivity analyses were carried out to assess the effect of potential data inaccuracies by reexamining low birth weight, preterm birth and SGA rates after restricting live births to those whose a) birth weight was measured on the day of birth, b) mothers were certain about the date of their last menstrual period and c) birth weight was not implausible for gestational age [27]. We also carried out logistic regression analyses to assess the effect of changes in maternal age (< 20, 20–24, 25–29, 30–34, 35–39 and ≥ 40 years), parity (0, 1–2 and ≥ 3), height (< 150, 150–154, 155–159 and ≥ 160 cms) and infant sex on temporal trends in preterm birth, low birth weight, SGA and perinatal mortality.

We determined etiologic fractions [28] for polychotomous categories of birth weight (< 1000, 1000–1999 and 2000–2499 g, with ≥ 2500 g as the reference). We also assessed the potential for reducing perinatal mortality through increased obstetric intervention and enhanced neonatal care by examining the rate of perinatal death among births with a birth weight ≥ 2,500 g (i.e., "preventable" deaths) [29]. Finally, we examined the influence of birth registration at the borderline of viability since regional and temporal contrasts of perinatal death can be biased because of differences in birth registration [30,31]. As per World Health Organization recommendations [32], we repeated our analyses of perinatal mortality after restricting regional and temporal contrasts to live births and stillbirths with a birth weight ≥ 1,000 g. We also graphed the gestational age-specific incidence of birth and perinatal death in the 2 regions using the fetuses at risk approach [33]. Under this approach, birth rates and perinatal death rates were calculated by dividing the number or births/perinatal deaths at any gestation by the fetuses at risk of birth/perinatal death at that gestation (i.e., those delivering at that gestation or beyond). Approval for the study was obtained from the Research Ethics Board of the IWK Health Centre, Halifax, Nova Scotia and from Christian Medical College, Vellore, Tamil Nadu.

## Results

There were 49,806 total births in Kaniyambadi and 208,020 total births in Nova Scotia between 1986 and 2005. The proportion of births to mothers < 20 years of age was 18.8% in Kaniyambadi and 7.6% in Nova Scotia. Births to older mothers, previous cesarean delivery rates and multiple birth rates were higher and previous perinatal death rates were lower in Nova Scotia (Table 1).

**Table 1:** Maternal and infant characteristics of all births in Kaniyambadi, South India and Nova Scotia, Canada, 1986 to 2005.

	Kaniyambadi		Nova Scotia	
	Number	Percent	Number	Percent
Total births	49,806	100.0	208,020	100.0
Maternal age				
< 20 years	9,364	18.8	15,715	7.6
20–24 years	25,469	51.1	47,010	22.6
25–29 years	12,069	24.2	70,775	34.0
30–34 years	2,264	4.6	53,466	25.7
35–39 years	581	1.2	18,461	8.9
≥ 40 years	57	0.1	2,593	1.3
Parity				
0	20,798	41.8	92,337	44.4
1–2	24,777	49.8	103,602	49.8
3–4	3,679	7.4	10,737	5.2
≥ 5	552	1.1	1,323	0.6
Previous cesarean delivery	1,220	2.5	22,093	10.6
Previous perinatal death	2,461	4.9	3,139	1.5
Multiple births	545	1.1	5,113	2.5

Subjects with missing information excluded from rate calculations

Births to teen mothers declined in both Kaniyambadi and in Nova Scotia, although absolute rates were much higher in Kaniyambadi (Table 2). There was little change in births to older mothers (≥ 35 years) in Kaniyambadi, whereas in Nova Scotia such births increased more than two-fold. The median maternal height in Kaniyambadi was 153 cm (mean 153.2, standard deviation 5.4 cm). The proportion of women who did not receive any antenatal care during pregnancy in Kaniyambadi declined from 6.2% in 1986–87 to 0.2% in 2004–05, while the proportion who had ≥ 3 antenatal visits increased from 67.2% in 1986–87 to 87.1% in 2004–05. Hospital deliveries increased from 41.0% in 1986–87 to 87.0% in 2004–05. Cesarean delivery rates increased from 3.1% in 1986–87 to 10.9% in 2004–05 in Kaniyambadi and from 20.2% to 28.4% in Nova Scotia.

**Table 2:** Temporal trends in maternal age, parity, operative vaginal delivery and cesarean delivery, Kaniyambadi (KB) and Nova Scotia (NS), 1986 to 2005.

Period	Age < 20 years (%)		Age ≥ 35 years (%)		Nulliparous (%)		Parity ≥ 5 (%)		Operative vaginal delivery rate (%)		Cesarean rate (%)	
						
	KB	NS	KB	NS	KB	NS	KB	NS	KB	NS	KB	NS
1986–87	25.1	7.6	1.9	6.3	39.4	43.9	2.4	0.6	3.4	13.4	3.1	20.2
1988–89	24.3	8.4	1.9	6.5	40.0	43.7	2.3	0.6	3.4	12.5	3.1	19.9
1990–91	21.9	8.7	1.5	7.6	37.8	44.5	1.5	0.6	5.0	11.0	3.9	18.8
1992–93	18.6	8.5	1.2	8.6	38.7	44.0	1.3	0.7	6.6	10.5	5.7	19.3
1994–95	18.4	8.5	1.1	9.4	40.4	44.3	0.9	0.6	4.8	10.6	6.1	19.4
1996–97	17.3	8.0	1.1	10.8	42.4	44.4	0.7	0.6	4.2	10.1	5.7	19.9
1998–99	16.9	7.6	1.2	12.3	44.6	44.5	0.6	0.8	3.5	10.6	6.5	20.4
2000–01	15.3	6.1	1.1	13.6	44.3	44.2	0.5	0.7	3.5	9.9	7.8	23.9
2002–03	14.5	5.7	0.7	14.8	44.2	45.2	0.3	0.6	4.0	10.7	8.2	27.7
2004–05	14.3	5.0	1.0	14.9	47.3	45.7	0.4	0.6	4.1	9.7	10.9	28.4

Total	18.8	7.6	1.3	10.1	41.8	44.4	1.1	0.6	4.2	10.9	6.0	21.4

**Figure 1:**
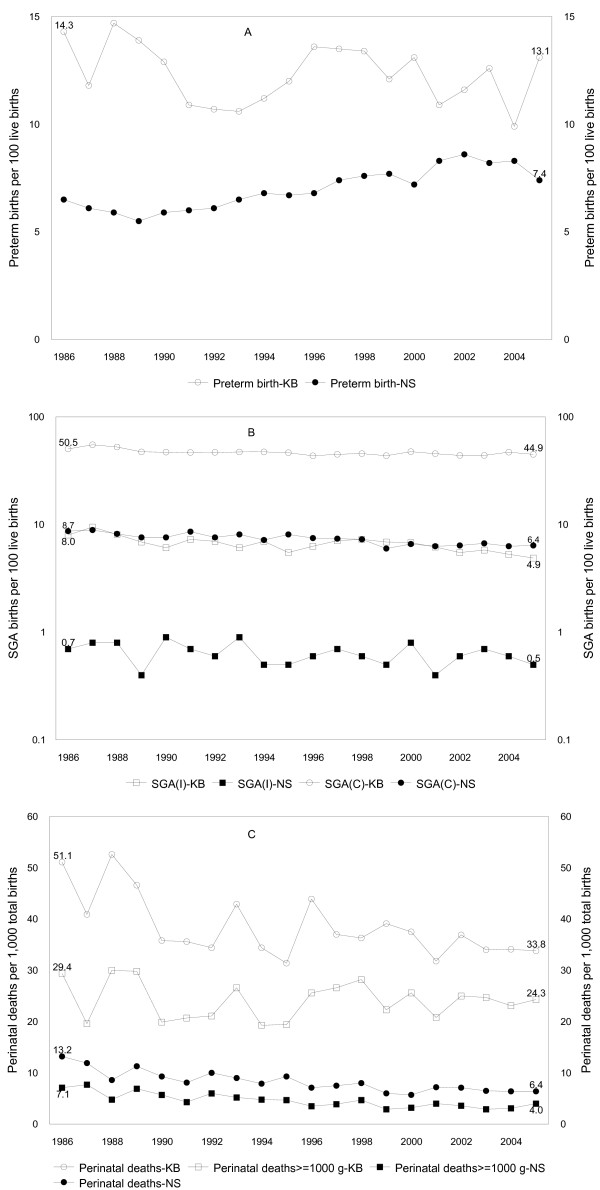
**Temporal trends in preterm birth (panel A), small-for-gestational age births (SGA, panel B) and perinatal mortality (panel C) in Kaniyambadi (KB) and Nova Scotia (NS), 1986 to 2005**. SGA-I refers to SGA births defined by an Indian fetal growth standard [26], while SGA-C refers to SGA births defined using a Canadian standard [25].

In Kaniyambadi, the overall low birth weight rate was 17.0% and the rate of preterm birth was 12.3%. The SGA rate was 46.9% when the Canadian fetal growth standard was used (Table 3) and 6.7% when the Indian standard was used to identify SGA infants. Low birth weight rates declined by 19% (95% confidence interval (CI)10–26%) between 1986–87 and 2004–05 (P for linear trend < 0.001). The decline was restricted to the early years; the trend from 1990–91 to 2004–05 was not significant (P = 0.37). There was a 12% (95% CI 2–21%) decrease in preterm birth but the the linear trend from 1990–91 onwards was not significant (P = 0.35, Figure 1). SGA rates declined by 13% (95% CI 9–17%) in terms of risk (and by 24% in terms of odds), from 52.8% in 1986–87 to 46.0% in 2004–05, when the Canadian fetal growth standard was used. SGA rates declined by 42% (95% CI 31–51%), from 8.8% in 1986–87 to 5.1%, in 2004–05 under the Indian standard; the linear trend was highly significant (P < 0.001), and the decline was not restricted to the early years (Table 3).

**Table 3:** Temporal trends in low birth weight, preterm birth, small-for-gestational age (SGA) birth and perinatal mortality rates, Kaniyambadi (KB) and Nova Scotia (NS), 1986 to 2005.

Period	Low birth weight/100 live births		Preterm births/100 live births		SGA rate/100 live births		Stillbirths/1,000 total births		Early neonatal deaths/1,000 live births		Perinatal deaths/1,000 total births	
						
	KB	NS	KB	NS	KB	NS	KB	NS	KB	NS	KB	NS
1986–87	18.9	5.8	13.0	6.3	52.8	8.8	23.2	8.0	23.3	4.5	46.0	12.6
1988–89	18.1	5.5	14.3	5.7	49.9	7.9	26.7	6.8	23.5	3.2	49.6	9.9
1990–91	16.2	5.6	11.9	6.0	47.0	8.1	18.1	5.7	17.9	3.1	35.7	8.8
1992–93	17.1	5.5	10.6	6.3	47.1	7.9	21.6	5.9	17.4	3.6	38.6	9.5
1994–95	16.1	5.5	11.6	6.7	46.9	7.7	18.0	5.5	15.2	3.1	32.9	8.6
1996–97	16.8	5.4	13.6	7.1	44.3	7.5	25.6	4.5	15.2	2.8	40.5	7.3
1998–99	18.2	5.2	12.7	7.7	44.6	6.7	20.9	5.0	17.2	2.0	37.7	7.0
2000–01	17.0	5.3	12.0	7.7	46.6	6.5	17.7	4.3	17.4	2.2	34.7	6.4
2002–03	16.0	5.5	12.1	8.4	43.9	6.6	17.5	4.8	18.3	2.0	35.5	6.8
2004–05	15.4	5.7	11.5	7.9	46.0	6.3	19.7	4.9	14.5	1.6	33.9	6.4

Total	17.0	5.5	12.3	6.9	46.9	7.5	20.9	5.6	18.0	2.9	38.6	8.5

Small for gestational age (SGA) rates determined using the 10th percentile cutoff of a Canadian fetal growth standard [25]. Perinatal outcome rates were estimated based on subjects without missing information (42,343 to 49,806 in Kaniyambadi and 159,893 to 208,020 in Nova Scotia).

Sensitivity analyses restricted to live births with more accurate data (58% of live births) did not substantially alter these indices (low birth weight in Kaniyambadi 17.4%, preterm birth 10.8% and SGA 48.0%). Regression analysis showed that adjustment for maternal age, parity, height and infant sex did not alter temporal trends in preterm birth and low birth weight. Preterm birth rates (2004–05 vs 1990–91) did not decline (adjusted odds ratio 1.00, 95% CI 0.89–1.14), while low birth weight rates showed a non-significant reduction (adjusted odds ratio 0.91, 95% CI 0.81–1.02, P = 0.11) in the adjusted models. SGA rates, based on the Canadian fetal growth standard, did not decrease significantly (adjusted odds ratio 0.93, 95% CI 0.85–1.01, P = 0.10), while SGA rates based on the Indian standard showed an attenuated, significant decline (adjusted odds ratio 0.73, 95% CI 0.61–0.88).

The rates of low birth weight (5.5%), preterm birth (6.9%) and SGA (7.5%) were substantially lower in Nova Scotia; the rates of these outcomes in the restricted subgroup with more accurate data (77% of live births) were 5.0%, 6.8% and 7.5%, respectively. The SGA rate in Nova Scotia was < 1% when the Indian fetal growth standard was used to identify SGA live births. Low birth weight rates were stable, preterm birth rates increased and SGA rates decreased in Nova Scotia between 1986–87 and 2004–05 (Table 3).

The perinatal mortality rate in Kaniyambadi was 38.6 per 1,000 total births compared with 8.5 per 1,000 total births in Nova Scotia. Stillbirth rates in Kaniyambadi decreased by 15% (95% CI -36% to +12%, P = 0.24) between 1986–87 and 2004–05; the linear trend between 1986–87 and 2004–05 was significant (P = 0.01) but that from 1990 onwards was not (P = 0.59). Early neonatal deaths decreased by 38% (95% CI 15–54%); the linear trend between 1986–87 and 2004–05 was significant (P = 0.002), although that from 1990–91 to 2004–05 was not (P = 0.66). Similarly, perinatal mortality rates declined by 26% (95% CI 10–40%) from 1986–87 to 2004–05; the linear trend over the entire study period was significant (P = 0.003), yet the trend from 1990 onwards was not (P = 0.49). Regression adjustment for maternal age, parity, height and infant sex did not change temporal trends in perinatal mortality rates; the adjusted odds ratio contrasting 2004–05 vs 1990–91 was 0.82 (95% CI 0.59–1.13). Rates of perinatal mortality in Nova Scotia declined by about 50%, with larger relative declines in early neonatal death rates (Table 3).

The perinatal mortality rate ≥ 1,000 g was 24.1 and 4.8 per 1,000 total births in Kaniyambadi and Nova Scotia, respectively (Figure 1). There was a non-significant 3% (95% CI -27% to +27%) decrease in perinatal death rates ≥ 1,000 g in Kaniyambadi between 1986–87 and 2004–05 (24.5 vs 23.7 per 1,000 total births, P for linear trend = 0.66). Perinatal deaths in Nova Scotia among births ≥ 1,000 g decreased by 52% (95% CI 35% to 64%, P < 0.001) from 7.4 per 1,000 total births to 3.6 per 1,000 total births.

The etiologic fraction calculations showed that in Kaniyambadi births < 1,000 g, 1,000–2,499 g and those with missing birth weight contributed to 6.6%, 27.6% and 35.6% of perinatal deaths, respectively, while in Nova Scotia these birth weight categories contributed 31.8%, 24.9% and 12.2%, respectively. Among births ≥ 2,500 g, the stillbirth rate in Kaniyambadi was 5.0 per 1,000 total births, the early neonatal death rate was 6.7 per 1,000 live births, the perinatal mortality rate was 11.7 per 1,000 total births and the cesarean delivery rate was 6.0%. In Nova Scotia, the same rates were 1.8 per 1,000 total births, 0.7 per 1,000 live births, 2.6 per 1,000 total births and 20.9%, respectively.

**Figure 2:**
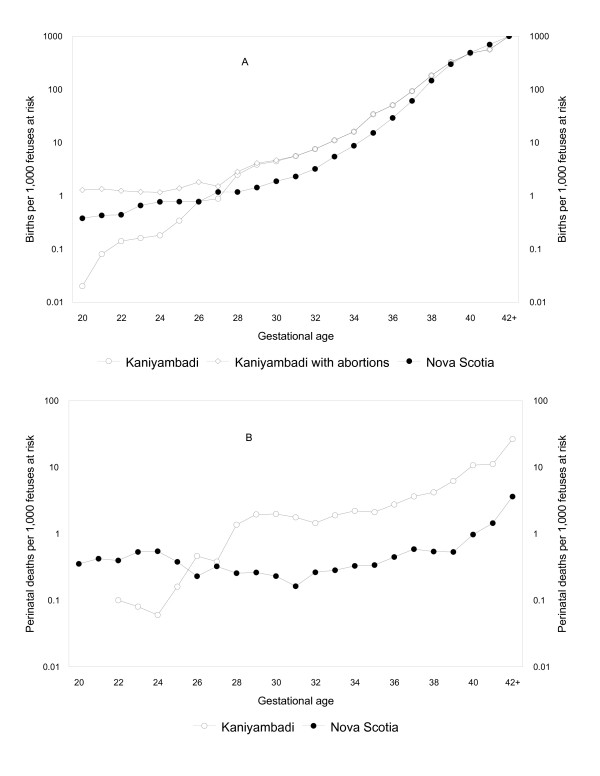
**Incidence of birth (panel A) and perinatal death (panel B) per 1,000 fetuses at risk in Kaniyambadi, South India and Nova Scotia, Canada, 1986 to 2005**. Birth rates in Kaniyambadi are also shown after including spontaneous abortions ≥ 20 weeks gestation.

**Figure 3:**
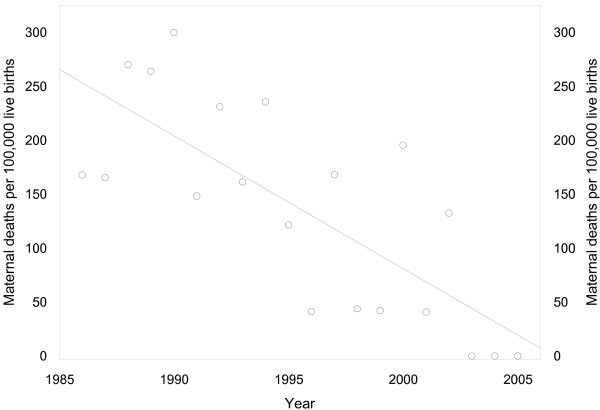
**Maternal mortality rates per 100,000 live births in Kaniyambadi, South India, 1986 to 2005 (line indicates linear trend)**.

Gestational age-specific birth and perinatal death rates in Kaniyambadi were several-fold higher than in Nova Scotia from 28 weeks onwards (Figure 2). Inclusion of spontaneous abortions (recorded in the Kaniyambadi database) increased the birth rates at < 28 weeks to a pattern consistent with birth and perinatal death rate differences observed at later gestation. The maternal mortality rate in Kaniyambadi decreased from 235.9 per 100,00 live births in 1986–90 to 35.5 per 100,000 live births in 2001–05 (85% decrease, 95% CI 57 to 95%; P for linear trend < 0.001, Figure 3).

## Discussion

Our study showed relatively high rates of preterm birth in a South Asian setting with high rates of low birth weight. SGA rates in this setting were dependent on the fetal growth standard used to define SGA, with very high rates observed when the Canadian fetal growth standard was used and low rates observed when an older Indian standard was used. A temporal decline was observed in SGA rates based on the Indian fetal growth standard but there was no significant change in low birth weight, preterm birth, SGA rates based on the Canadian standard and perinatal mortality rates between 1990 and 2005. Similarly, perinatal mortality rates among those with birth weights ≥ 1,000 g did not show any reduction between 1986–87 and 2004–05. Rates of preventable perinatal death (i.e., those among births ≥ 2,500 g) were high and cesarean delivery rates were low in Kaniyambadi relative to Nova Scotia, suggesting one potential avenue for reducing perinatal mortality [29]. Birth registration in Kaniyambadi was incomplete prior to 28 weeks of gestation (Figure 2). On a positive note, maternal mortality rates declined substantially in Kaniyambadi between 1986 and 2005, presumably as a consequence of an increase in hospital births.

SGA rates were very high in Kaniyambadi under the Canadian fetal growth standard and low under the decades-old Indian fetal growth standard. The low 10^th ^percentile cut-off of the Indian standard (e.g., at 40 weeks the mean birth weight was 2,892 g, the standard deviation was 451 g and the 10^th ^percentile was 2,315 g) resulted in a SGA rate of 6.7% which was substantially lower than the low birth weight rate (17%). This suggests that the Indian standard [26] is perhaps better viewed as a descriptive (rather than normative) standard. Controversies surrounding the need for customized fetal growth standards notwithstanding [34-36], research needs to be directed at developing an appropriate fetal growth standard for Indian live births. Recent developments in this area, specifically methods that allow the creation of outcome-based fetal growth standards [37], may prove helpful in developing a consensus on an appropriate standard for Indian fetuses.

We could not use a more recent South Indian fetal growth standard [38] because of the restricted gestational age range studied and because actual percentiles were not provided in the publication. Use of this more recent standard [38] would have resulted in higher rates of SGA compared with the older Indian standard (e.g., the 10^th ^percentile at 40 weeks was 2,315 g in the older Indian standard, and approximately 2,550 g for males and 2,450 g for females according to the South Indian standard [38]). Regression adjustment for maternal age, parity, height and infant sex attenuated the temporal decline in SGA rates suggesting that some of the improvement in fetal growth was due to changes in these factors. The adjusted 27% decline in SGA rates (observed under the Indian standard [26]), though encouraging, has to be set against the absence of an appreciable decline in low birth weight rates.

The relatively high rate of preterm birth observed in Kaniyambadi also requires attention; preterm birth is increasingly being recognized as a primary contributor to perinatal death in less industrialized countries [39]. The use of antenatal steroids and surfactant is not common in less industrialized countries, though some of these technologies may be sufficiently cost-effective to be deployed widely if appropriate infrastructure and adequately trained personnel are available [14].

Infant mortality rates in India and other less industrialized countries appear to show large reductions in recent years (from 84 in 1990 to 56 per 1,000 live births in 2005 in India [1]). Whereas the progress is laudable, the absolute rates probably represent a fraction of all infant deaths. As observed in Kaniyambadi, which has a relatively high-quality health information system, birth registration is pragmatic (rather than definition-based) and births < 28 weeks are less likely to be registered than in Nova Scotia. Such variations are also seen when Canada is compared with some European countries [40].

Our study showed no significant changes in preterm birth, low birth weight and perinatal mortality from 1990 to 2005. It is possible that the higher rates of several perinatal outcomes in the first few years of program (1986–89) represent a reporting artefact. The failure to reduce low birth weight, preterm birth and perinatal death rates in the 16 years after 1990 raises the question of health policy focus With nutritional supplementation having minimal efficacy in increasing gestational weight gain and birth weight [41] and no available means for substantial prevention of preterm birth, it may be appropriate to consider increasing the emphasis on community and hospital-based infrastructure and personnel training to reduce infant morbidity and mortality [14,42]. The experience of industrialized and less industrialized countries in reducing infant mortality through reductions in birth weight- and gestational age-specific mortality (including reductions in deaths among normal birth weight births [10-12]) also lends credence to this position.

The above-mentioned need to reconsider the health policy focus (from reducing low birth weight to reducing birth weight-specific mortality) is also supported by the experience of immigrant Asian-Indians. Studies show that Asian-Indians in the United States have better socio-demographic risk profiles (e.g., lower rates of teen mothers, higher socioeconomic status, better prenatal care), higher rates of preterm birth, much higher rates of small-for-gestational age, higher rates of fetal death and the same (or modestly higher) neonatal and post-neonatal mortality rates as their white counterparts [43-45]. These findings suggests that improved socioeconomic conditions and concomitant improvements in nutrition and prenatal care may not succeed in preventing growth restriction and attendant intrauterine problems in this population. On the other hand, rates of fetal, neonatal and postneonatal mortality among Asian-Indians in the United States are substantially lower than those observed in Kaniyambadi because of existing differences in health care services.

The relatively high rates of perinatal death and the relatively low rates of cesarean delivery among births ≥ 2,500 g also support the need to enhance the availability and uptake of health care services over the medium and long term future. The absence of a change in preterm birth rates in Kaniyambadi also reflects a health service issue; iatrogenic preterm birth at 34–36 weeks gestational age has increased in industrialized settings and this has been associated with declines in perinatal death [46,47]. Whereas the overall cesarean delivery rate in Kaniyambadi (10.9% in 2004–05) falls within the WHO recommendation [48], it is highly questionable whether a 10–15% cesarean delivery rate is appropriate for a population with such a high level of intrauterine morbidity and mortality. On the other hand, the provision of a higher intensity of health services will require the development of obstetric and neonatal care infrastructure and personnel. Also, prevention of perinatal death by such intervention would only be appropriate if nutritional, infectious and other causes of infant death are simultaneously controlled in the population. Finally, attempts to increase rates of cesarean delivery and other obstetric intervention without appropriate infrastructure and trained personnel could have adverse effects with regard to maternal and infant morbidity and mortality. Nevertheless, the increase in hospital deliveries from 41.0% in 1986–87 to 87.0% in 2004–05 suggests that this rural population is seeking a higher level of obstetric and neonatal care.

The applicability of our study's findings to other parts of India and related environments deserves comment. Kaniyambadi has intensive community- and hospital-based health care services and other inputs that exceed those extant in other parts of rural and semi-rural India. Therefore some of the findings of our study may not directly apply to regions without such services. Nevertheless, we believe our study provides some important caveats for reducing perinatal mortality rates in India and similar countries. Intensive community-based and other inputs, although successful in reducing post-neonatal mortality, are unlikely to reduce rates of preterm birth, low birth weight and perinatal death in such populations. Therefore, a focus on reducing low birth weight may not be as productive as a focus on reducing perinatal morbidity and mortality across the birth weight range through investments in health care infrastructure and personnel.

The strengths of our study include a longstanding, prospective collection of data through a closely monitored health information system. Data were collected by trained personnel who worked closely with village-resident health workers, thereby ensuring completeness and accuracy of the information. Although Kaniyambadi has a relatively small population, it is likely representative of other rural populations in south India. Our study has several limitations, however. Primary among these is the potential for data errors in various measurements including birth weight and menstrual-based gestational age. Menstrual-based estimates of gestational age suffer from a higher degree of error than gestational age confirmed by early ultrasound measurements [49,50]. However, it is noteworthy that routine reports from industrialized countries based on vital statistics data show substantially different rates of preterm birth compared to preterm birth rates that are based on the best clinical estimate of gestation [51,52]. For instance, the preterm birth rate in the United States was 12.2% and 10.1% in 2002 when gestational age was based on menstrual dates and the best clinical estimate, respectively [51]. Also, the preterm birth rate in routine vital statistics reports from Britsh Columbia, Canada was approximately 30% lower than the same rate based on an algorithm combining menstrual and early ultrasound information (7.5% vs 9.7% in 2005 [52]). For this reason we used gestational age based on menstrual dates for both Nova Scotia and Kaniyambadi. One study (on an albeit dissimilar population of women seeking early abortion) has shown that the accuracy of women's estimates of pregnancy duration (based on menstrual dates) were no different in Pune, India compared with Atlanta, United States [53]. Other limitations of our study included a lack of information on some important determinants of perinatal outcome (e.g., prepregnancy weight) and problems with estimating gestational age and birth weight among stillbirths. In Nova Scotia, women whose menstrual-based gestational age differed from the clinical estimate were assigned a missing menstrual-based gestational age. Infants of such women had lower birth weights than other infants, which probably explains the lower-than-expected SGA rates in Nova Scotia (Table 3). The absence of information regarding the timing (antepartum vs intrapartum) and cause of stillbirths in Kaniyambadi is another limitation of our study.

## Conclusion

Our study showed high rates of low birth weight and relatively high rates of preterm birth in Kaniyambadi. The magnitude of the SGA rates was dependent on the fetal growth standard used to define SGA, with very high rates observed when the Canadian fetal growth standard was used and low rates observed when an older Indian standard was used. Secular declines in SGA were also dependent on the fetal growth standard used. No substantial declines were observed in low birth weight, preterm birth, or perinatal death over the previous 16 years, whereas maternal mortality rates declined dramatically. Overall, this picture suggests the need for a reexamination of the traditionally focus on preventing low birth weight and for increasing support for clinical infrastructure and trained personnel.

## Competing interests

The authors declare that they have no competing interests.

## Authors' contributions

This study was carried out by the Christian Medical College-Canada Collaborative Group, which includes all the listed authors. KSJ proposed the study. Methods and preliminary analyses were discussed by all the authors (KG, JP, DS, SM, DA, JM, KSJ, JJ and MSK) and other staff at CHAD hospital. Additional analyses were carried and a manuscript was drafted by KSJ. This was revised for intellectual content based on comments from the authors and all authors approved the final version of the manuscript.

## Pre-publication history

The pre-publication history for this paper can be accessed here:


